# Teratoma-associated anti-NMDAR encephalitis: Two cases report and literature review

**DOI:** 10.1097/MD.0000000000009177

**Published:** 2017-12-08

**Authors:** Zhigang Liang, Shaowan Yang, Xuwen Sun, Bing Li, Wei Li, Zhuli Liu, Guoping Yu

**Affiliations:** a Department of Neurology, Yantai Yuhuangding Hospital Affiliated to Qingdao University, Yantai, Shandong; b Department of Neurology, Beijing Tiantan Hospital, Beijing, P. R. China.

**Keywords:** anti-NDMA receptor, autoimmune encephalitis, immune therapy, teratoma

## Abstract

**Objective:**

This study aimed to discuss the pathogenesis, clinical manifestation, diagnosis, and treatment of anti-*N*-methyl-D-aspartate receptor (NMDAR) encephalitis.

**Case Report:**

The diagnosis and treatment of 2 cases with teratoma-associated anti-NMDAR encephalitis were summarized and the clinical data of patients reported by domestic and international studies were reviewed in this study. The 2 cases were both adolescent females who showed mental abnormalities as their main clinical manifestation. The patients were positive for anti-NMDAR antibody in their serum and cerebrospinal fluid, and gynecologic ultrasound detected ovarian teratoma. After diagnosis, the patients underwent teratoma resection, followed by pulse therapy of hormones and gamma globulin. Chemotherapy was performed to prevent tumor recurrence, and patients were in a stable condition.

**Conclusions:**

Teratoma-associated anti-NMDAR encephalitis is commonly seen in young women. The clinical manifestation of this disease is nonspecific, and the patients mainly have fever, psychosis, and seizure. Tumor resection and immune therapy are effective treatment strategies, and standardized chemotherapy should also be performed to prevent recurrence.

## 1 Introduction

Anti-*N*-methyl-D-aspartate receptor (NMDAR) encephalitis is an autoimmune encephalitis that is associated with the NMDA receptor and has a good response to treatment.^[1,2]^ The disease was first named by Dalmau et al^[2]^ in 2007, and related studies have been reported successively in the following years. Due to the lack of clinical practice guidelines for the prevention and treatment of anti-NMDAR encephalitis, this study aimed to summarize the clinical data of 2 patients who had teratoma-associated anti-NMDAR encephalitis and received treatment in Department of Neurology, Yantai Yuhuangding Hospital Affiliated to Qingdao University and also to review recent studies for more detailed recognition of this disease.

### 1.1 Case report

#### 1.1.1 Patient 1: Female adolescent, aged 17 years, hospitalization No. 114

The patient was first admitted on November 23, 2015, due to “fever for a week, dizziness, and disorganized speech for 2 days.” On November 16, 2015, the patient had a fever, pharyngeal pain, and a body temperature of 37.5°C. Seven days later, the patient showed aggravated symptoms of excitement, speech confusion, and unresponsiveness, and was therefore sent to Department of Neurology, Yantai Yuhuangding Hospital Affiliated to Qingdao University. Past history revealed that the patient underwent excision of right ovarian teratoma a year ago. She had a negative menstrual history, personal history, and family history.

Physical examination on admission showed body temperature 37.4°C, pulse 73 beats/min, respiration 18 times/min, and blood pressure 112/76 mm Hg. The patient was conscious but unresponsive, dull in expression, and showed impaired ability of calculating and memorizing. The neurological examination showed no positive sign.

**Figure 1:**
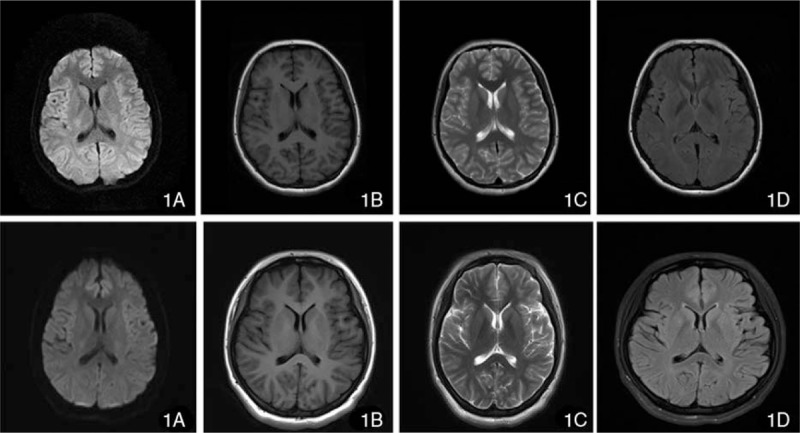
(1A–1D) Craniocerebral MRI scanning of patient 1. DWI, T1, T2, and Flare images showed no abnormalities. (2A–2D) Craniocerebral MRI scanning of patient 2. DWI, T1, T2, and Flare images showed no abnormalities.

Accessory examinations showed the routine blood test, which suggested a total white blood cell (WBC) count of 10.72 × 10^9^/L. The lumbar puncture showed a cerebrospinal fluid (CSF) pressure of 170 mmH_2_O; the routine CSF test suggested a WBC count of 50 × 10^6^/L, with 90% mononuclear cells; and the biochemical test indicated normal CSF biochemistry. No abnormality was identified from tests of C-reactive protein, blood coagulation, procalcitonin, stool + occult blood, or erythrocyte sedimentation rate (ESR). Neither craniocerebral-enhanced magnetic resonance imaging (MRI) (Fig. 1 1A–1D) nor chest x-ray, electrocardiogram (ECG) showed any abnormalities, and electroencephalogram (EEG) showed slow wave. On December 4, 2015, further examination demonstrated NMDA-R-Ab (+) in the CSF and NMDA-R-Ab (–) in the serum. Therefore, the patient was diagnosed with anti-NMDAR encephalitis. The patient was given pulse therapy of gamma globulin (25 g/day) and methylprednisolone (1 g/day) for 5 days and later turned to oral administration of drugs. After treatment, the condition of the patient improved and she was discharged on December 23, 2015.

**Figure 2:**
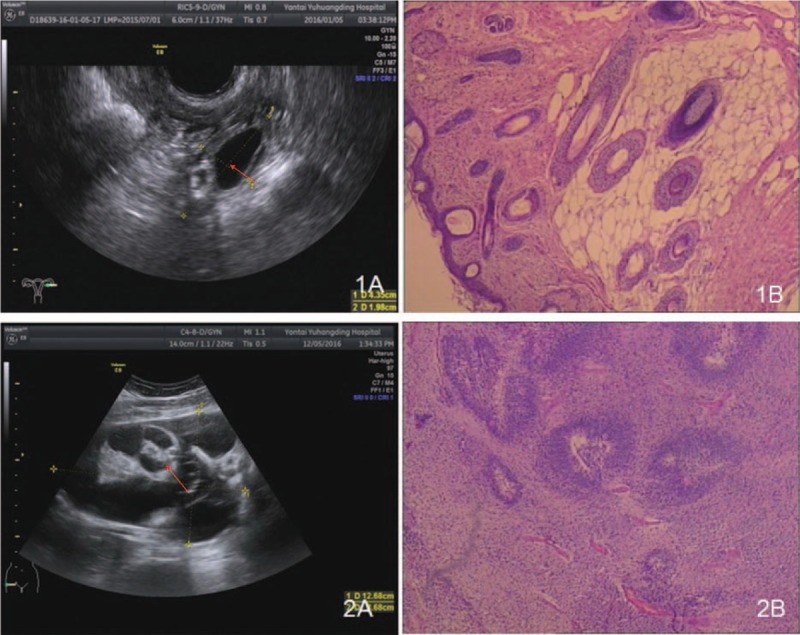
Patient 1: (1A) Ultrasound detected a hyperechoic nodule in the left ovary, with a size of approximately 1.4 × 1.2 cm^2^ (red arrow) (1B) The pathological examination revealed a cystic-solid nodule, with a wall thickness of 0.2–0.3 cm.

**Figure 3:**
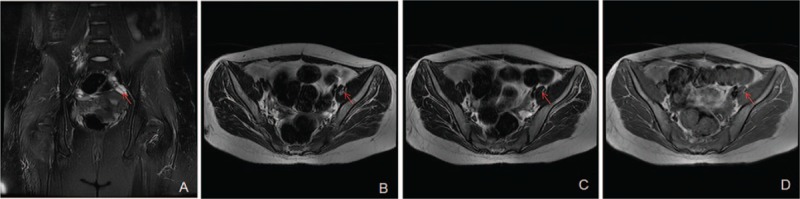
In patient 1, MRI scanning detected a patchy lesion with short T1 and long T2 signal change on the posterior side of the left ovary (red arrow); the fat suppression image showed a low signal with long T1 and short T2 in the center. The size of the lesion was approximately 0.6 × 0.9 cm^2^, and the T2-weighted fat suppression image showed a local signal reduction. After teratoma excision, the right ovary had a clear image of follicles, and no abnormal signal was detected.

On December 24, 2015, the patient was again hospitalized. Physical examination on admission revealed that the patient was conscious but dull in expression, and had repetitive speech. The ability of calculating, memorizing, and understanding was impaired. The patient showed positive Romberg sign (+). Limb strength was normal. Accessory examinations showed that the routine blood test suggested a WBC count of 23.09 × 10^9^/L, with 86.4% neutrophils. Gynecological ultrasound (Fig. 2 1A) detected a hyperechoic nodule in the left ovary, with a size of approximately 1.4 × 1.2 cm^2^. Pelvic-enhanced MRI (Fig. 3) revealed a patchy lesion with short T1 and long T2 on the posterior side of the left ovary; the fat suppression image showed a low signal with long T1 and short T2 in the center. The size of the lesion was approximately 0.6 × 0.9 cm^2^, and the T2-weighted fat suppression image showed a local signal reduction. After teratoma excision, the right ovary had a clear image of follicles, and no abnormal signal was detected. On the basis of these results, the patient was considered to have recurrent teratoma.

Treatment of the patient was given immunotherapy [gamma globulin (25 g/day) and methylprednisolone (1 g/day) for 5 days and later turned to oral administration of drugs], and her mental symptoms improved afterward. On January 8, 2016, the patient was anesthetized with tracheal intubation and received laparoscopic enucleation of the left ovarian tumor performed by a gynecological chief physician. With general anesthesia + abdominal fascia block anesthesia method, general anesthesia was maintained with 0.375% ropivacaine (40 mL), Propofol injection (500 mg), Cisatriptan for injection (23 mg), Fentanyl citrate injection (20 μg), Remifentanil (1 mg). The patient's ECG, noninvasive blood pressure, pulse oximetry, body temperature, and Bispectral Idex (BIS) were monitored. The patient's intraoperative systolic blood pressure was 90 to 140 mm Hg. During the operation, it was observed that the cyst had a diameter of about 1.5 cm, smooth surface, and no adhesion to peripheral tissues. No abnormalities were found in the left fallopian tube or right appendages. Intraoperative fast pathology suggested cystic mature teratoma (Fig. 2 1B). Immunotherapy was continued after the operation, and 3 months later, chemotherapy was performed, with an intramuscular injection of bleomycin (25 mg) on Day1 and Day2, intravenous injection of cisplatin (50 mg) on Day3 and Day4, and intravenous injection of etoposide (0.16 g) from Day1 to Day3. After 4 courses of chemotherapy, the patient was discharged and followed up for 1 year. She was in a stable condition during the follow-up.

### 1.2 Patient 2: Female adolescent, aged 16 years, hospitalization no. 131

The patient was admitted on December 2, 2016, due to “psychotic disorder and paroxysmal convulsion for 20 days.” On November 10, 2016, the patient showed abnormal behavior and confused speech at school. Two days later, she developed limb twitching and slow reaction, was unable to urinate or defecate by herself, did not eat, had a low fever, and the body temperature did not reach above 38°C. The patient had a negative menstrual history, personal history, past history, and family history.

Physical examination on admission revealed body temperature 37.2°C, pulse 80 beats/min, respiration 19 times/min, and blood pressure 138/56 mm Hg. The patient was in a stuporous state, did not respond to questions, and showed no spontaneous speech. Her body was in waxy flexibility, and the limbs showed high muscle tension. The Babinski and Chaddock signs were negative.

**Figure 4:**
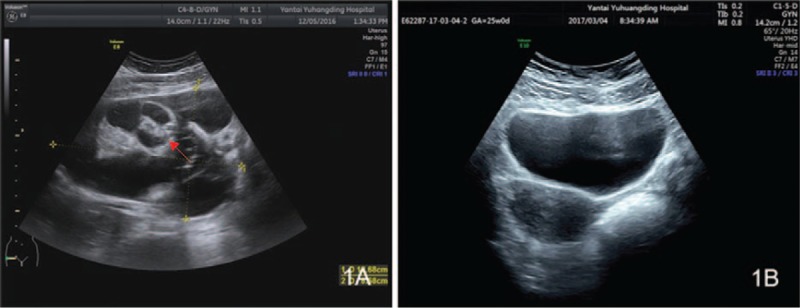
Patient 2: (1A) Before teratoma resection, a mix-echoic mass (12.7 × 12.5 × 8.8 cm^3^) (red arrow) could be seen in the right anterior uterus, with its upper boundary reaching to the umbilical level. Inside the mass, a multilocular septal echo with uneven thickness could be seen, and the cyst fluid showed good sound permeability. (1B) After teratoma resection, the left ovary was 3.6 × 2.2 cm^2^ in size, and no mass was observed.

Accessory examinations showed the blood test suggested a WBC count of 17.67 × 10^9^/L, with 78.9% neutrophils. The concentration of C-reactive protein was 16.3 mg/L, and the ESR was 66 mm/h. Tumor examination showed that the level of squamous cell carcinoma antigen, alpha-fetoprotein, and carbohydrate antigen 12-5 was 1.9 ng/mL, 54.13 ng/mL, and 60.37 U/mL; these tumor marker levels are abnormal, respectively. No abnormalities were found for the test of TORCH (Toxoplasmagondii, Toxo, Rubellavirus, Cytomegalovirus, CMV), Herpes simplex virus, HSV), or respiratory virus (*Legionella pneumophila* LP, *Mycoplasma pneumonia* MP, Q fever Rickettsia QX, *Chlamydia pneumonia* CP, adenovirus AAV, respiratory syncytial virus RSV, influenza A IFA, influenza B IFB, and parainfluenza viruses 1, 2, and 3 PIVS 9 items), nor did the patient show abnormal results for the test of thyroid function (triiodothyronine thyroid hormone, thyroxine, free triiodothyronine, freethyroxine thyroglobulin antibody 6 items). Both ECG and enhanced brain MRI (Fig. 1 2A–D) indicated normal results, but EEG showed high-amplitude θ waves in the slow waves. The ultrasound examination of the bladder and uterine appendages detected a mix-echoic mass, with a size of 12.7 × 12.5 × 8.8 cm^3^, in the right anterior uterus. The upper boundary of the mass reached the umbilical level, and multilocular septal echo with uneven thickness could be seen in the mass. No definite papillae were observed in the cyst wall, and the cyst fluid showed good sound permeability (Fig. 2 2A). The ultrasound examination of the liver, gallbladder, pancreas, spleen, and kidney did not identify significant abnormalities. The lumbar puncture showed that the CSF pressure was 190 mmH_2_O; CSF biochemistry indicated that the level of glucose and CSF protein was 4.42 mmol/L and 131.2 mg/L, respectively; the routine CSF test and CSF bacteriology did not suggest any significant abnormalities. The CSF was positive for NMDA-R-Ab (+), 1:100; the serum was negative for NMDA-R-Ab (–); and both the CSF and serum were negative for contactin associated protein-like 2 antibodies (CASPR2-Ab) and leucine-rich glioma inactivated protein 1 antibodies (LGI1-Ab) Anti-myeloperoxidase *antibody* 1-R-Ab, Anti-myeloperoxidase *antibody* 2-R-Ab, and gamma aminobutyric acid B-R-Ab. On the basis of the aforementioned results, viral encephalitis^[3]^ and psychiatric sickness^[4]^ were excluded, and the patient was diagnosed with anti-NMDAR encephalitis. On December 14, 2016, the patient was anesthetized by tracheal intubation and underwent right appendectomy performed by a gynecological chief physician. General anesthesia was maintained with Midazolam (2 mLa), Etomidate (20 mg), Cisatriptan (14 mg), Fentanyl (0.3 mg), Propofol (500 mg), and Remifentanil (1 mg). During the operation, the patient's ECG, noninvasive blood pressure, pulse oximetry, body temperature, and BIS were monitored. The patient's intraoperative systolic blood pressure was 90 to 130 mm Hg. During the surgery, a multilocular cystic tumor was found in the pelvic cavity; the tumor was 13 × 13 × 10 cm^3^ in size, irregular in shape, and was encapsulated by an integrated envelope. The tumor was believed to originate from the right ovary, and the left appendages and right fallopian tube were normal in appearance. Intraoperative fast pathology indicated an ovarian high-grade immature teratoma (Grade 2–3) (Fig. 2 2B**)**. Therefore, the patient was diagnosed with teratoma-associated anti-NMDAR encephalitis. After the operation, the patient was given the pulse therapy of gamma globulin (20 g/day) for 5 days, followed by the pulse therapy of methylprednisolone (500 mg/day), and the dose was decreased gradually. The patient was in a stable condition afterward and received teratoma chemotherapy at the Department of Gynecology after 1 month. After gynecological ultrasound review (Fig. 4 1B), PEB (platinum, etoposide, bleomycin) chemotherapy was performed with an intramuscular injection of bleomycin (25 mg) on D1 and D2, intravenous injection of cisplatin (50 mg) on D3 and D4, and intravenous injection of etoposide (0.16 g) from D1 to D3. The patient was followed up for 3 months, and she was in a stable condition during this period.

This study was approved by the ethics committee of Yantai Yuhuangding Hospital Affiliated to Qingdao University. The family members of both patients signed their informed consent after being explained regarding the treatment and operation plan.

## 2 Discussion

NMDAR encephalitis was first reported by Vitaliani et al^[5]^ in 2005. Two years later, Dalmau et al^[2]^ named the disease as anti-NMDAR encephalitis and suggested it to be caused by the specific interaction between an autoantibody and the NMDAR 1 NR1 subunit of NMDAR. In recent years, domestic and international studies successively reported individual cases of anti-NMDAR encephalitis, but exact epidemiological data are still lacking. In this study, two cases who received treatment in Yantai Yuhuangding Hospital Affiliated to Qingdao University were analyzed, and also the clinical data of patients with teratoma-associated anti-NMDAR encephalitis reported by previous studies were reviewed.

A large number of studies have reported anti-NMDAR encephalitis in recent years.^[1,6–8]^ It is believed that this disease is closely associated with the development of a tumor, particularly teratoma. The NMDAR, a heteromer consisting of 2 subunits NR1 and NMDAR 1NR2, is mainly distributed in the hippocampus, prefrontal cortex, amygdala, and hypothalamus, and is involved in a higher nervous activity. The anti-NMDAR antibody interacts with the epitope located at the end of NR1 and can induce the internalization of NMDAR, thereby impairing the synaptic function mediated by NMDAR.^[9]^ The impaired synaptic function would reduce the inhibition on postsynaptic glutamate transmitters and further increase the release of glutamate from subcortex and prefrontal cortex, finally resulting in schizophrenic symptoms and movement disorders. Previous studies found that NMDAR antagonists (ketamine, for example) could cause symptoms similar to those of anti-NMDAR encephalitis,^[10]^ whereas NMDAR agonists alleviated schizophrenic symptoms.^[11]^ It is believed that patients with teratoma-associated anti-NMDAR encephalitis may have nerve components in their tumors.^[12,13]^ Among all adult patients with teratoma, the incidence of anti-NMDAR encephalitis was as high as 59%, indicating that the relationship between anti-NMDAR encephalitis and teratoma might be a part of antitumor reaction.^[1]^ Or, under the background of infection, the immune system might stimulate the body to generate certain autoantibodies to react with the NMDAR of neurons, and these autoantibodies might exhibit ectopic expression in the ectodermal tissue of teratoma.^[14,15]^

Anti-NMDAR encephalitis has characteristic clinical manifestations. The major symptoms include prodromal symptoms such as fever, headache, vomiting, and diarrhea^[7]^; mental symptoms such as anxiety, paranoia, fear, and mania (adolescent females are particularly prone to mental abnormalities and catatonic schizophrenia^[16,17]^); and body symptoms such as seizures, cognitive disorders, autonomic dysfunction, and sleep difficulties. The course of diseases can be divided into 5 stages: the prodromal stage, mental symptom stage, nonresponsive stage, hypermotility stage, and recovery stage.^[18]^ The characteristics of patients included in the present study were as follows: First, Patient 1 showed fever before disease onset and exhibited mental symptoms 4 days after disease onset. Patient 2 also showed fever and exhibited psychosis, twitching, and slow reaction in the early phase of the disease, which corresponded to the clinical manifestation of anti-NMDAR encephalitis. Second, craniocerebral MRI scanning showed no significant abnormalities in both patients during their hospital stay. Third, EEG is useful in anti-NMDAR encephalitis. In a study addressing the EEG features of 23 anti-NMDAR encephalitis patients, the main findings were diffuse background slowing with delta slow waves and generalized extreme delta brush.^[19]^ Generalized rhythmic delta activity in anti-NMDAR encephalitis may represent the effect of the antibodies against the NMDAR, leading to reduced NMDA function. Of the 2 patients in this study, the first EEG had only slow waves and the second had EEG a slow wave: The whole figure is irregular in waveform. The long-range and short-range medium-high amplitude 5 to 7 c/s θ waves are active. Fast wave: Each show scattered short-range low amplitude 15 to 18 c/s rhythm and activity. Because we did not perform dynamic EEG testing, we found no more noticeable abnormalities. Anti-NMDAR encephalitis in patients with EEG also showed no specific changes; although sometimes not only slow wave, but also completely normal, the study by Schmitt et al^[19]^ and other studies have shown that abnormal δ brush may be anti-NMDAR encephalitis on the EEG Of the specific performance, mainly seen in the longer course and the condition of the heavier patients.^[20]^ The 2 patients with EEG showed only abnormal, slow wave, but no specific performance. Fourth, the detection of anti-NMDAR antibody in the serum and CSF was key to the diagnosis of anti-NMDAR encephalitis. In the present study, both patients had an increased level of anti-NMDAR antibody. Therefore, they were diagnosed with anti-NMDAR encephalitis. Together with the present cases, this study reviewed a total of 14 cases of teratoma-associated anti-NMDAR encephalitis (Table 1 ). Out of the 14 cases, 13 were adolescent females, and their clinical manifestations included mental abnormalities and seizures. Nerve components were found in the teratoma of 6 patients; positive anti-NMDAR antibody was identified for 13 patients either in their CSF or serum; EEG and MRI suggested no abnormalities; and teratoma resection in combination with immune therapy was carried out for 13 patients, with 12 cases of good prognosis and 1 case of death. In the present case report, patient 1 developed encephalopathy symptoms after left teratoma resection, and the diagnosis was recurrent anti-NMDAR encephalitis caused by teratoma recurrence. Patient 2 underwent teratoma resection, followed by immune therapy of gamma globulin and hormones, and was given chemotherapy after the symptoms improved. Thus, for patients with teratoma-associated anti-NMDAR encephalitis, tumor recurrence should be prevented by performing standardized chemotherapy and follow-up. In the past literature, most patients with NMDA receptor encephalitis recovered or remained residual, but some suffered serious defects and died. After the serum antibody titer is reduced, it may take at least a year to improve.^[21]^ The prognosis of patients with our reported cases and retrospective domestic literature is basically the same.

**Table 1:**
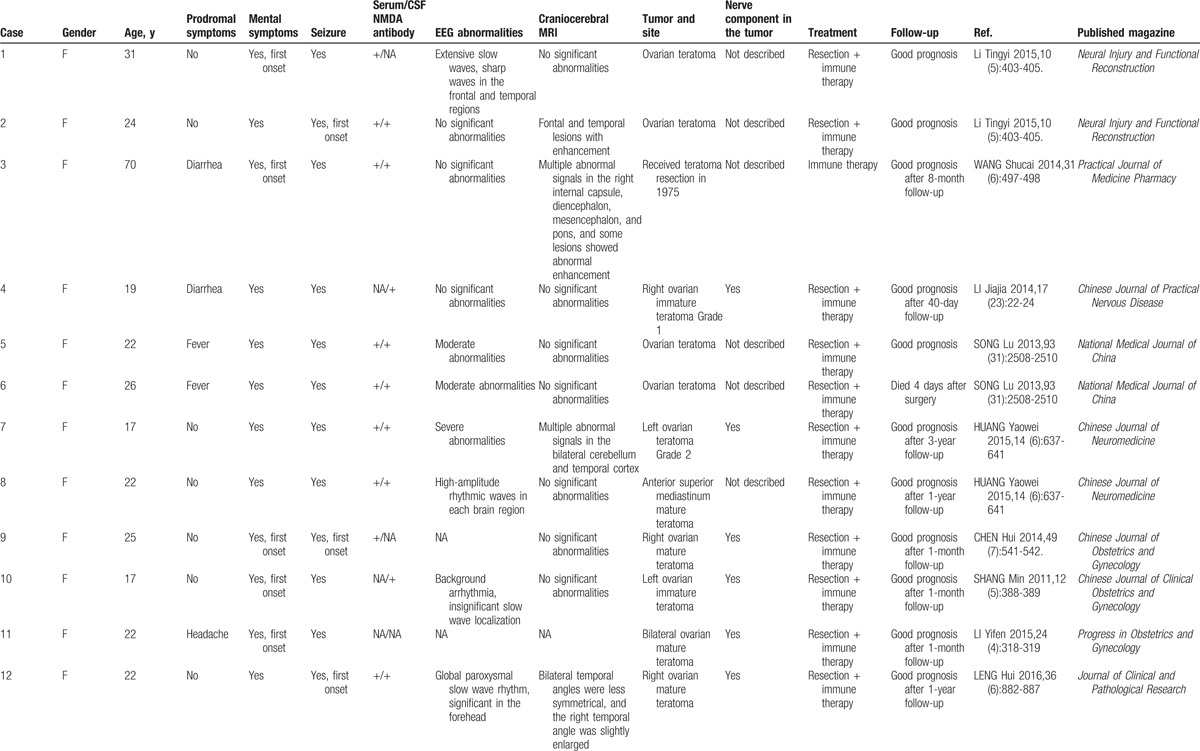
Fourteen cases of teratoma-associated anti-NMDAR encephalitis from the literature review.

**Table 1 (Continued):**
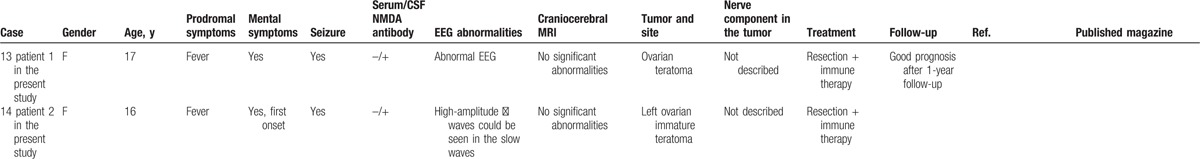
Fourteen cases of teratoma-associated anti-NMDAR encephalitis from the literature review.

The treatment of anti-NMDA encephalitis mainly includes tumor resection and immune therapy,^[13]^ and tumor resection is particularly a key to treatment. It is also recommended that controversial anesthetics, which likely act at NMDARs, should be avoided. Anesthetics alter the activation of the central nervous system by excitatory or excitatory inhibitory neurons. NMDAR mediates excitatory neurotransmission in the nervous system. A variety of electrophysiological studies have shown that ketamine and nitrous oxide have an inhibitory effect on NMDARs, so these patients should be avoided. In this article, two patients were performed an operation under induce and maintain anesthesia. The mechanism by which inhaled anesthetics and propofol are nebulized is not well understood and there is no definitive conclusion about the effects of NMDAR or GABA A receptors on inhaled anesthetics and propofol. Numerous studies show that propofol anesthesia enhances GABAergic delivery. GABA A receptors play an important role in inducing anesthesia. This report uses propofol, fentanyl, and rizatriptan to induce and maintain anesthesia in 2 patients. Ideally, controversial narcotics that could potentially act on NMDARs should be avoided.^[22,23]^

The first-line immune therapies mainly include the use of gamma globulin, glucocorticoids, or plasma substitution, and second-line immune therapies mainly include single or combined use of rituximab and cyclophosphamide. In the present case report, patient 1 underwent teratoma resection 1 year ago, but tumor recurrence again caused anti-NMDAR encephalitis because standardized chemotherapy was not performed before the first teratoma resection. This suggests that for teratoma-associated anti-NMDAR encephalitis, chemotherapy should be performed to prevent tumor recurrence. Despite no recommended clinical guideline, studies recommend female patients (aged more than 12 years) to undergo abdominal and pelvic MRI scanning every half year in the first 4 years after treatment.^[24]^ As for patient 2, gynecological ultrasound and abdominal MRI identified an immature teratoma, and the patient was given standardized chemotherapy after teratoma resection. For the treatment of adolescent females with teratoma-associated anti-NMDAR encephalitis, the suggestions include the following: gynecologic oncology consultation should be performed to determine an effective treatment plan for the teratoma; and after teratoma resection, the patient should undergo regular gynecologic ultrasound or pelvic computed tomography/MRI scanning to prevent recurrence.

In conclusion, for female patients showing symptoms similar to those of virus encephalitis, such as psychosis or seizures, the possibility of anti-NMDAR encephalitis should be considered, and the detailed screening of tumor antibodies and anti-NMDAR antibodies should be performed. Anti-NMDAR encephalitis is a common autoimmune encephalitis.^[25]^ As for the clinical diagnosis and treatment of this disease, detailed tumor screening and pathological examination should be performed, and detection of anti-NMDAR antibodies should also be carried out. Standardized immune therapy, tumor resection, postoperative chemotherapy, and follow-up would help to prevent the incidence of tumor recurrence.
